# Environmental detection of eumycetoma pathogens using multiplex real-time PCR for soil DNA in Sennar State, Sudan

**DOI:** 10.1186/s41182-023-00563-3

**Published:** 2023-12-19

**Authors:** Hiroki Hashizume, Suguru Taga, Masayuki K. Sakata, Mahmoud Hussein, Emmanuel Edwar Siddig, Toshifumi Minamoto, Ahmed Hassan Fahal, Satoshi Kaneko

**Affiliations:** 1 School of Tropical Medicine and Global Health, Nagasaki University, 1-12-4 Sakamoto, Nagasaki, 852-8523 Japan; 2 Department of Ecoepidemiology, Institute of Tropical Medicine (NEKKEN), Nagasaki University, 1-12-4 Sakamoto, Nagasaki, 852-8523 Japan; 3 Graduate School of Human Development and Environment, Kobe University, 3-11 Tsurukabuto, Nada-Ku, Kobe, 657-8501 Japan; 4 Research Faculty of Agriculture, Hokkaido University, Kita-9, Nishi-9, Kita-Ku, Sapporo, Hokkaido 060-8589 Japan; 5 Mycetoma Research Center, University of Khartoum, P.O. Box 102, Khartoum, Sudan; 6 Tumors Therapy and Cancer Research Center, Molecular Biology Unit, Shendi University, P.O .Box 142-143, Shendi, Sudan; 7 Faculty of Medical Laboratory Sciences, Unit of Basic Medical Sciences, University of Khartoum, Khartoum, Sudan

**Keywords:** Mycetoma, *Madurella*, *Falciformispora*, Environmental DNA, Soil, Diagnosis

## Abstract

**Background:**

Mycetoma is a chronic disease affecting the skin and subcutaneous tissue endemic in the tropical and subtropical regions. Several bacteria and fungi can cause mycetoma, but fungal mycetoma (eumycetoma) is challenging because the treatment requires a combination of a long-term antifungal agent and surgery. Although the transmission route has not yet been elucidated, infection from the soil is a leading hypothesis. However, there are few soil investigation studies, and the geographical distribution of mycetoma pathogens is not well documented. Here, we used multiplex real-time PCR technology to identify three fungal species from soil samples.

**Methods:**

In total, 64 DNA samples were extracted from soil collected in seven villages in an endemic area in Sennar State, Sudan, in 2019. Primers and fluorescent probes specifically targeting the ribosomal DNA of *Madurella mycetomatis*, *Falciformispora senegalensis*, and *F. tompkinsii* were designed.

**Results:**

Multiplex real-time PCR was performed and identified the major pathogen, *M. mycetomatis* that existed in most sites (95%). In addition, two other pathogens were identified from some sites. This is the first report on the use of this technique for identifying the eumycetoma causative microorganisms.

**Conclusions:**

This study demonstrated that soil DNA investigation can elucidate the risk area of mycetoma-causative agents. The results will contribute to the design of prevention measures, and further large-scale studies may be effective in understanding the natural habitats of mycetoma pathogens.

**Supplementary Information:**

The online version contains supplementary material available at 10.1186/s41182-023-00563-3.

## Introduction

Mycetoma is a persistent inflammatory disease that primarily affects the subcutaneous layers of the skin. It is commonly found in tropical and subtropical regions, but cases have been reported worldwide. It tends to affect individuals residing in impoverished and isolated communities [1–4]. Mycetoma is characterized by the formation of several painless masses caused by inflammation. These masses tend to produce a discharge that contains grains and is typically seropurulent in nature. If left untreated, the disease can progress and invade not only the skin but also deeper structures and even bones [5, 6]. Mycetoma has been classified into two types according to the causative organisms: actinomycetoma, caused by groups of bacteria, and eumycetoma, caused by fungi. In Sudan, the country with the highest number of reported cases globally, eumycetoma is responsible for more than 70% of the cases. The common culprits behind these infections include *Madurella mycetomatis*, *Falciformispora senegalensis,* and *F. tompkinsii* [7–10].

While actinomycetoma can be effectively managed with a combination of antibiotics, treating eumycetoma with antifungals poses significant challenges [11, 12]. A few drugs are currently used for eumycetoma treatment; however, none of them are able to eradicate pathogenic fungi completely. As a result, extensive surgical excisions and, in severe cases, limb amputations may be required as a part of the treatment process [10, 13].

Despite the first case of eumycetoma being reported in India over 150 years ago, there is still a lack of comprehensive understanding regarding the disease’s fundamental epidemiological characteristics. This knowledge gap presents a significant challenge in formulating effective strategies for prevention. Without a clear understanding of how the disease spreads, identifying high-risk populations and implementing targeted prevention measures becomes difficult [11, 14–16]. Furthermore, the infection typically progresses painlessly and slowly during the initial stages, which often leads to delayed medical consultations and diagnosis of advanced disease [17, 18]. As a result, crucial information pertaining to the disease, such as its prevalence, incidence, incubation period, and entry route, remains largely unclear. The lack of timely medical intervention and limited data on these crucial epidemiological factors contribute to the challenges in understanding and effectively managing mycetoma.

Although the transmission path has not been identified, a prevailing theory suggests subcutaneous inoculation of causative organisms from the soil through minor skin wounds caused by trauma or plant thorns [11, 19]. Many individuals living in endemic regions walk barefoot or use open footwear such as sandals, putting them at a higher risk due to their increased susceptibility to minor injuries [13, 20]. Therefore, understanding the geographical distribution of the causative fungi on the ground would be necessary to bridge the epidemiological knowledge gap [21, 22]. Several studies attempted to isolate or detect the mycetoma pathogeny by collecting soil samples in endemic areas [19, 23]. Also, other studies using environmental DNA (eDNA) extracted from soil with molecular identification technology have been reported [19, 24–26]. However, large-scale environmental studies on fungal mycetoma were not reported. Furthermore, no study specifically targeted eumycetoma pathogens other than *M. mycetomatis*. In this study, we present the results of the first soil investigation to identify multiple eumycetoma pathogens in Sudan using multiplexed real-time PCR, a highly sensitive detection method targeting the prime agent, *M. mycetomatis,* and two other pathogens (*F. senegalensis* and *F. tompkinsii*) to establish a process that becomes the first step toward determining the modes of transmission of mycetoma as well as to increase experimental efficiency of the measurements.

## Methods

### Soil sampling

**Fig. 1:**
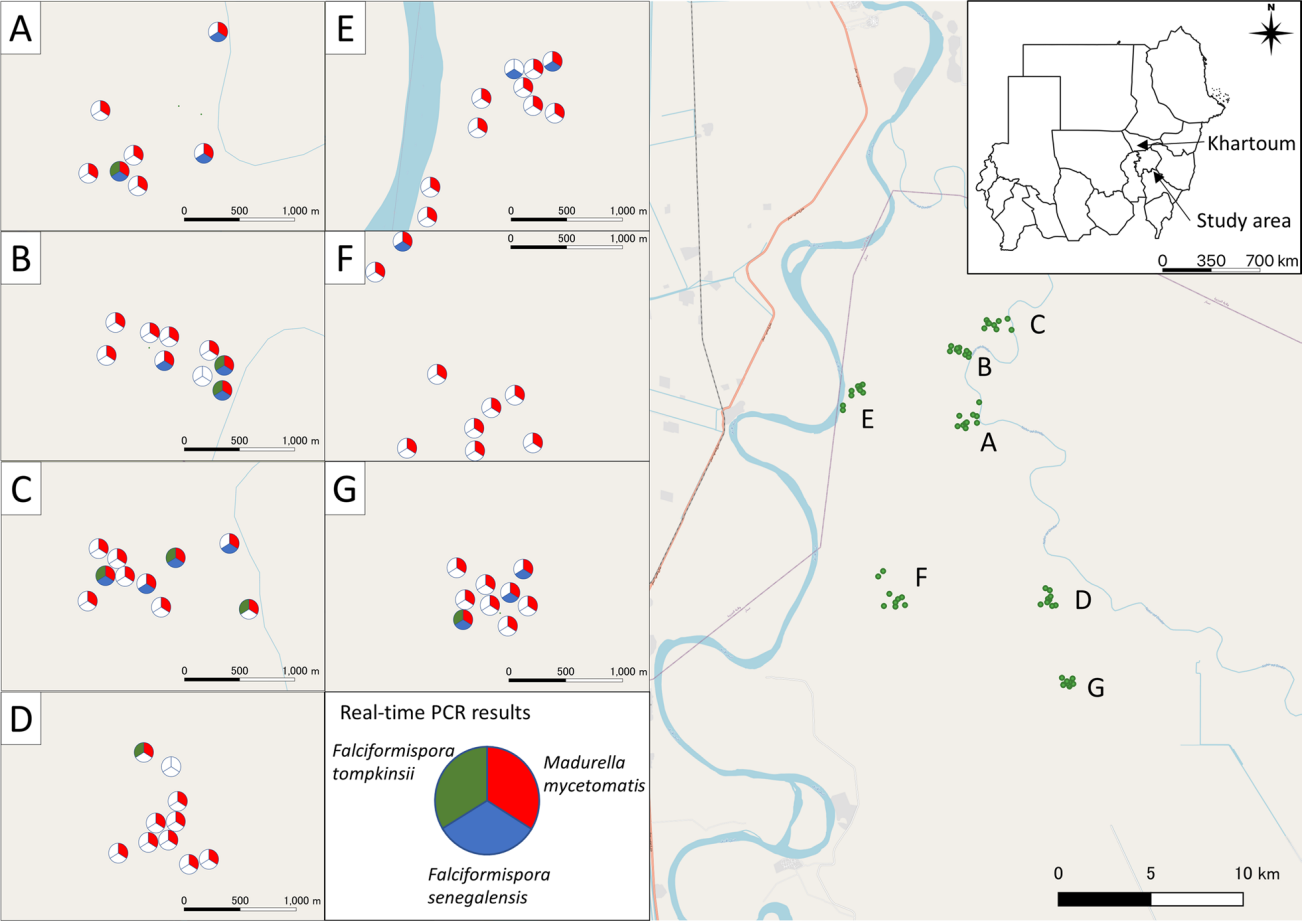
Map of sampling location with the result of multiplex PCR. Each point on the map presents a sample collection site. Letters refer to the sampling villages: **A** Deshein, **B** Sharfa Al-Mihrab, **C** Al-Awia, **D** Wad Hassan, **E** Wad Ajeeb, **F** Al-Ragal Al-Batahin and **G** Wad Al-har. Pie charts indicate the detection of the target species: red color: *Madurella mycetomatis*; blue color: *Falciformispora senegalensis*; and green color: *Falciformispora tompkinsii*. Map data were downloaded from the OpenStreetMap project (OpenStreetMap contributors) under a CC BY-SA 2.0 license (www.openstreetmap.org). The map was created using the QGIS Geographic Information System, Open Source Geospatial Foundation Project, under a CC BY-SA 3.0 license (http://qgis.osgeo.org)

**Fig. 2:**
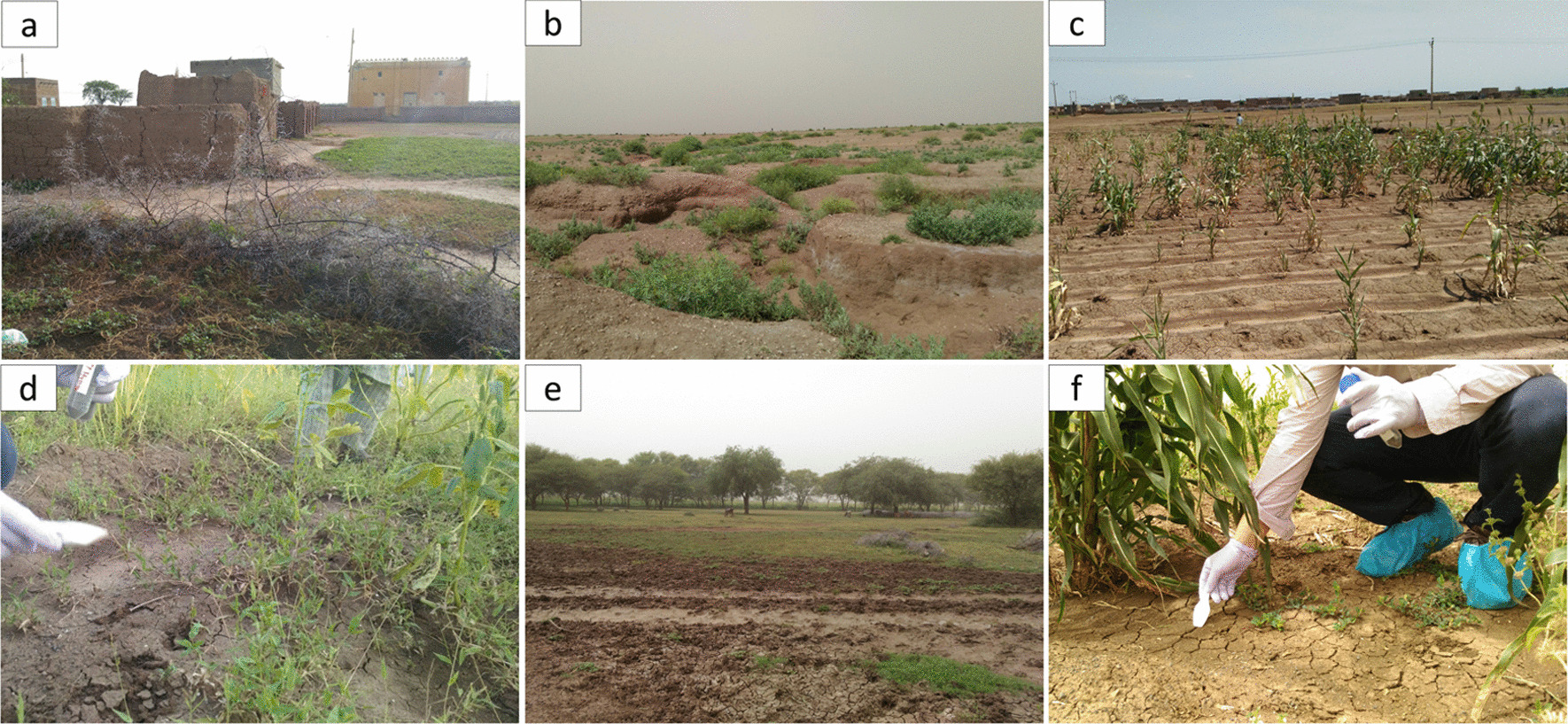
Five categories of sampling sites and soil collection. Letters correspond to representative sampling sites by category and soil sampling: **a** cattle grazing area, **b** dryland, **c** farm, **d** riverside farm, **e** road and **f** soil sampling

The study area was located in the north of Sennar State, Sudan, approximately 250 km southeast of the capital city of Khartoum, in a hot desert climate (Fig. 1). It is an endemic area for eumycetoma and has a clinic run by the MRC. Soil samples were collected for eDNA analysis, a highly sensitive and rapid method of species identification from DNA in environmental samples. The detailed sampling protocol was described in the previous study [26]. Briefly, soil samples were collected and extracted from 64 sites from October 16 to 17, 2019. Ten sampling sites per village were chosen from seven villages (Fig. 1). The land usage categorized each sampling site in the field (cattle grazing area, dryland, farmland, riverside farm, and road) (Fig. 2) [26]. Soils were sampled from the ground surface into a 50-ml plastic centrifuge tube using disposable plastic shovels while wearing disposable gloves and shoe covers against DNA contamination (Fig. 2). Soils in plastic tubes were kept on ice during the sampling and in a freezer on return to the laboratory. Alkaline DNA extraction with ethanol precipitation and a commercial kit for soil was conducted in the MRC laboratory (PowerSoil DNA Isolation Kit, Qiagen, Germany) [27, 28]. Negative controls were obtained using 9 g of distilled water for each DNA extraction in village units.

### Primer and probe design

Species-specific primers and probes were designed to target Sudan’s three most common pathogens. Sequences of the ribosomal DNA of *M. mycetomatis*, *F. senegalensis*, *F. tompkinsii* and other eumycetoma pathogens were downloaded from the National Center for Biotechnology Information (NCBI: https://www.ncbi.nlm.nih.gov) [26]. These sequences were aligned using Unipro UGENE (version 40.0), and then primers and probes for the three species were developed in the ribosomal DNA ITS1 region (Table 1). The primers contain two-base target-specific substitutions within five bases from their 3′ end. The designed primer sets were analyzed using Primer3 software [29–31] to consider melting temperature. Then, they were checked in silico using NCBI Primer-BLAST search (https://www.ncbi.nlm.nih.gov/tools/primer-blast/) against the NCBI nucleotide collection database (nt) with default settings to test the primer specificity [32]. The test on the primer sets estimated sole amplification of each target species, suggesting sufficient specificity for eDNA analysis. Subsequently, the cross-reactivity of each primer/probe set was checked in vitro using three target synthetic genes (Integrated DNA Technologies, USA). Each 20 µl reaction contained 3 × 10^0^ − 10^3^ copies of the synthetic gene solution, 500 nM of each primer set, and 125 nM of each probe in the TaqMan Environmental Master Mix 2.0 (ThermoFisher Scientific, USA). The thermal conditions were as follows: 2 min at 50 °C, 10 min initial denaturation at 95 °C, and 55 cycles of 15 s denaturing at 95 °C and 1 min annealing at 60 °C. These PCR amplifications were performed using the Bio-Rad CFX96 Real-time PCR system (Bio-Rad, USA). As a result of the in vitro test by multiplex real-time PCR, the fluorescence of each target DNA was detected, and no cross-reactivity was observed.

**Table 1:** List of the oligonucleotides used in this study

Target species	Primer/probe	Sequence^a^	Product size
*Madurella mycetomatis*	M.m_forward	5′-CCTCCCGGTAGTGTAGTGTCC-3′	89 bp
	M.m_reverse	5′-GAGAGGCCGTACAGAGCAAAT-3′	
	M.m_probe	5′-FAM-GGCGTCCGCCGGAGGATTATACAAC-BHQ1-3′	
*Falciformispora senegalensis*	F.s_forward	5′-GTTCCTACGCCGGCAAC-3′	119 bp
	F.s_reverse	5′-AGACAGGTATACTGCTTTTGCTGC-3′	
	F.s_probe	5′-HEX- GCCGCTGGGTCTCCACC-BHQ1-3′	
*Falciformispora tompkinsii*	F.t_forward	5′-CTTTGGCTCTGCCACTGC-3′	123 bp
	F.t_reverse	5′-GACAAGTGTACTGCTTCTAACGGC-3′	
	F.t_probe	5′-TAMRA- TCTGCCGCTGGGCATCTTAAT -BHQ2-3′	

^a^FAM: fluorescein amidite, BHQ: Black Hole Quencher, HEX: hexachloro-fluorescein, TAMRA: carboxytetramethyl rhodamine

### PCR amplification

Multiplex real-time PCR using the soil eDNA samples of Sennar state in Sudan was conducted. Each 20 µl reaction contained 2 µl of eDNA template, 500 nM of each primer set, and 125 nM of each probe in the TaqMan Environmental Master Mix 2.0 (ThermoFisher Scientific). The thermal conditions were as follows: 2 min at 50 °C, 10 min at 95 °C, and 55 cycles of 15 s at 95 °C and 1 min at 60 °C. Three DNA sample replicates were amplified for each eDNA sample, 3 × 10^0^ − 10^3^ copies of the synthetic gene of the target fungi, and PCR negative control. During the evaluation process, the relative fluorescence unit (RFU) threshold was manually set to 200 and amplification curves were visually checked to remove errors. Because the eDNA samples might contain PCR inhibitors, target eDNA was considered detected if any one of the three replicates of each sample was positive, rather than quantitative evaluation. Since the eDNA of *M. mycetomatis* was detected from many sites, one-third of the total amplicons were sequenced. For the other two fungi, all amplicons were sequenced.

## Results

With the multiplex real-time PCR on field soil samples, the eDNA of three target fungi was successfully amplified (Fig. 1 and Additional file 1: Table S1). No amplicon was found from negative controls of DNA extraction or the PCR preparation process. The *R*^2^ values of the standard curve for the multiplex assay ranged from 0.966 to 0.998 (Additional file 1: Table S2). All sequenced samples were identified as the target species in the BLAST results.

**Fig. 3:**
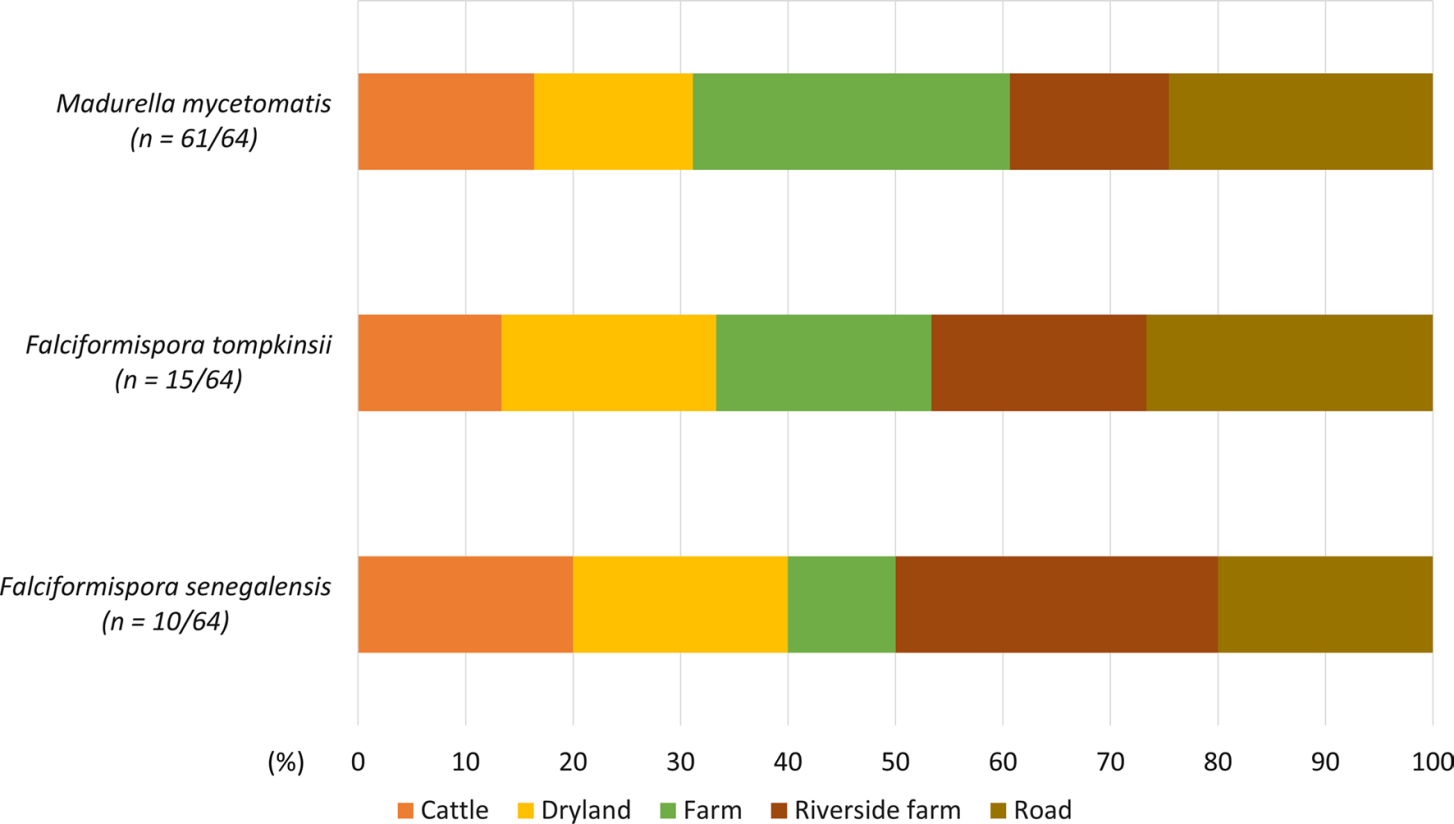
The result of real-time PCR targeting the three pathogens of eumycetoma. Each bar chart shows the rates of land use where the target species were detected

The prime causative agent of eumycetoma, *M. mycetomatis*, was identified in 61 of 64 soil samples (95%). The eDNA of *F. senegalensis* and *F. tompkinsii* was detected in 10 (16%) and 15 (23%) soil samples, respectively. All three species were detected in five land-use categories, suggesting that the causative agents are ubiquitous around villages in endemic areas (Fig. 3).

## Discussion

In this study, we demonstrated the efficiency of the multiplex real-time PCR, a highly sensitive molecular analysis method, for identifying eumycetoma causative organisms in soils of different land uses in an endemic area. Additionally, this marks the first application of this technique for identifying the eumycetoma pathogenic microorganisms. Notably, this technique detected two other organisms in the soil for the first time.

The investigation revealed that nearly all soil eDNA samples contained the DNA of the eumycetoma prime agent, *M. mycetomatis* (95%). In contrast, *F. senegalensis* and *F. tompkinsii* were identified only in 16% and 23% of soil samples, respectively. Although *M. mycetomatis* was expected to be present in a wide range of soils in the endemic area, such a high detection rate is remarkable both epidemiologically and ecologically. The results will help understand and address issues of eumycetoma. Previous reports have predicted mycetoma risk areas using climatic data, soil type, and *Acacia* distribution as indicators [21, 22]. The eDNA data could also be used as a novel environmental indicator for estimating mycetoma distribution.

The Mycetoma Research Centre (MRC), the University of Khartoum, WHO Collaborating Centre on Mycetoma, the main reference center in the country, which was established in Sudan in 1991, provides medical care and research on mycetoma. According to the MRC patient records, *M. mycetomatis* was the most common specimen isolated and identified in 70% of the patients, followed by actinomycetoma pathogens (*Streptomyces somaliensis* and *Actinomadura madurae*) [8, 10]. *F. senegalensis* and *F. tompkinsii* are occasionally isolated from patients but are uncommon pathogen species [10, 18]. Also, the study carried out in the nearby area by another group in Sudan, which conducted molecular identification from black-grain specimens from patients, presented similar results, *M. mycetomatis* was the predominant agent (88.2%) [33]. The similarity between the percentages of sites where the causative organisms were detected in the soil and the causative organisms seen in mycetoma patients would be potentially useful data.

The data obtained in this study provided corroborative evidence that mycetoma causative agents are prevalent in the soil of an endemic area in any land use. Thus, reducing contact with sharp objects and soil by wearing shoes regularly is essential to avoid minor skin injuries and wounds and the implantation of mycetoma-causative organisms into subcutaneous tissue [11]. Nonetheless, further research is needed to determine the route and risk factors for mycetoma.

The *M. mycetomatis* detection rate in the soil samples differs from the conventional PCR methods reported by Ahmed and colleagues [19], which was 23% (17 out of 74) and the metabarcoding method reported by Hashizume et al*.* was 23% (15 out of 64) [26]. It is noteworthy that real-time PCR can detect the excitation of fluorescence probes with high efficiency compared to the conventional PCR method. There are two explanations for the superiority of the real-time PCR detection rate compared to the metabarcoding method in mycetoma; first, the enzyme solution mix for environmental specimens was used in real-time PCR. The TaqMan Environmental Master Mix 2.0 reagent can amplify the target DNA in the presence of a high level of PCR inhibitors [34]. Also, the PCR enzymes were selected for metabarcoding with the priority to high fidelity for sequencing. PCR reagent differences may have led to a gap in the detection rates. Second, in real-time PCR, the primer set was explicitly targeted to *M. mycetomatis*, while in the metabarcoding method, the universal primers amplify any fungal DNA, most of which are not pathogens. Therefore, DNA reads of eumycetoma pathogens were relatively reduced, which induced lower sensitivity. In terms of highly sensitive identification, real-time PCR-based analysis yielded superior results in the three fungi, including *F. senegalensis* and *F. tompkinsii*. Overall, while metabarcoding has the advantage of detecting a large number of species, both major and minor causative microorganisms, real-time PCR has the strength of identifying targets with higher sensitivity.

Multiplex real-time PCR technique has been broadly applied for eDNA studies [35, 36]. Adding fluorescent probes can detect five or more species in one PCR test depending on instruments of real-time PCR systems. Therefore, multiplex real-time PCR targeting mycetoma pathogens could be a practical method with robust specificity and sensitivity for epidemiological surveys, diagnosis, and preventive and control measures.

## Conclusions

Real-time PCR eDNA analysis can be widely used, as it is a highly sensitive technology that could detect three pathogens of eumycetoma from the soil. Furthermore, the study first revealed that the significant eumycetoma agent, *M. mycetomatis*, was prevalent in the soil of the endemic area. Applying this technique to determine the geographic distribution of the mycetoma-causative microorganisms in the soil would provide fundamental data to bridge the knowledge gap in mycetoma epidemiology, diagnosis, and preventive measures.

### Supplementary Information

**Additional file 1: Table S1.** The result of real-time PCR targeting the three top pathogens of eumycetoma. The rates in the table present PCR positives in three replicates. **Table S2.** Summary of standard curves of three fluorescent.

## Data Availability

Data and materials used to conduct this study are available on request.

## Abbreviations

eDNA: Environmental DNA
RFU: Relative fluorescence unit
