# Supplementary material for: Environmental detection of eumycetoma pathogens using multiplex real-time PCR for soil DNA in Sennar State, Sudan
Source: Trop Med Health. 2023 Dec 19;51:71. doi: 10.1186/s41182-023-00563-3 (PMC10729560; doi:10.1186/s41182-023-00563-3)
Supplement: Supplementary file 1 — Additional file 1: Table S1. The result of real-time PCR targeting the three top pathogens of eumycetoma. The rates in the table present PCR positives in three replicates. Table S2. Summary of standard curves of three fluorescent. [file 41182_2023_563_MOESM1_ESM.docx]

**Table S1. The result of real-time PCR targeting the three top pathogens of eumycetoma**

The rates in the table present PCR positives in three replicates.

| Village | Land use | *Madurella*  *mycetomatis* | *Falciformispora*  *senegalensis* | *Falciformispora*  *tompkinsii* |
| --- | --- | --- | --- | --- |
| A. Deshein | Farm 1 | 3/3 | 0/3 | 0/3 |
|  | Farm 2 | 3/3 | 0/3 | 0/3 |
|  | Road | 3/3 | 2/3 | 1/3 |
|  | Cattle 1 | 3/3 | 0/3 | 0/3 |
|  | Cattle 2 | 2/3 | 0/3 | 0/3 |
|  | Dryland | 3/3 | 1/3 | 0/3 |
|  | Riverside farm | 3/3 | 0/3 | 0/3 |
| B. Sharfa Al-Mihrab | Farm 1 | 1/3 | 0/3 | 0/3 |
|  | Cattle | 3/3 | 0/3 | 0/3 |
|  | Road 1 | 3/3 | 0/3 | 0/3 |
|  | Dryland 1 | 2/3 | 0/3 | 0/3 |
|  | Riverside farm 1 | 2/3 | 3/3 | 3/3 |
|  | Riverside farm 2 | 3/3 | 3/3 | 3/3 |
|  | Dryland 2 | 0/3 | 0/3 | 0/3 |
|  | Road 2 | 3/3 | 2/3 | 0/3 |
|  | Farm 2 | 3/3 | 0/3 | 0/3 |
| C. Al-Awia | Farm 1 | 3/3 | 0/3 | 0/3 |
|  | Farm 2 | 3/3 | 0/3 | 0/3 |
|  | Cattle 1 | 1/3 | 0/3 | 0/3 |
|  | Road 1 | 3/3 | 0/3 | 0/3 |
|  | Road 2 | 3/3 | 3/3 | 3/3 |
|  | Cattle 2 | 3/3 | 1/3 | 0/3 |
|  | Dryland 1 | 3/3 | 2/3 | 2/3 |
|  | Riverside farm 1 | 3/3 | 3/3 | 0/3 |
|  | Riverside farm 2 | 3/3 | 0/3 | 1/3 |
|  | Dryland 2 | 3/3 | 0/3 | 0/3 |
| D. Wad Hassan | Farm 1 | 3/3 | 0/3 | 0/3 |
|  | Riverside farm 1 | 3/3 | 0/3 | 0/3 |
|  | Cattle 1 | 3/3 | 0/3 | 0/3 |
|  | Road 1 | 3/3 | 0/3 | 0/3 |
|  | Road 2 | 3/3 | 0/3 | 0/3 |
|  | Cattle 2 | 3/3 | 0/3 | 1/3 |
|  | Farm 2 | 3/3 | 0/3 | 0/3 |
|  | Dryland 1 | 3/3 | 0/3 | 1/3 |
|  | Dryland 2 | 0/3 | 0/3 | 0/3 |
|  | Riverside farm 2 | 3/3 | 0/3 | 0/3 |
| E. Wad Ajeeb | Farm 1 | 3/3 | 1/3 | 0/3 |
|  | Farm 2 | 3/3 | 0/3 | 0/3 |
|  | Road 1 | 1/3 | 0/3 | 0/3 |
|  | Cattle 1 | 0/3 | 1/3 | 0/3 |
|  | Road 2 | 2/3 | 0/3 | 0/3 |
|  | Cattle 2 | 3/3 | 0/3 | 0/3 |
|  | Dryland 1 | 2/3 | 0/3 | 0/3 |
|  | Dryland 1 | 2/3 | 0/3 | 0/3 |
|  | Riverside farm 1 | 3/3 | 0/3 | 0/3 |
|  | Riverside farm 2 | 3/3 | 0/3 | 0/3 |
| F. Al-Ragal Al-Batahin | Dryland 1 | 2/3 | 2/3 | 0/3 |
|  | Dryland 2 | 1/3 | 0/3 | 0/3 |
|  | Farm 1 | 3/3 | 0/3 | 0/3 |
|  | Farm 2 | 2/3 | 0/3 | 0/3 |
|  | Cattle | 3/3 | 0/3 | 0/3 |
|  | Road 1 | 3/3 | 0/3 | 0/3 |
|  | Road 2 | 3/3 | 0/3 | 0/3 |
|  | Farm 3 | 3/3 | 0/3 | 0/3 |
|  | Farm 4 | 3/3 | 0/3 | 0/3 |
| G. Wad Al-har | Road 1 | 3/3 | 0/3 | 0/3 |
|  | Farm 1 | 3/3 | 1/3 | 1/3 |
|  | Farm 2 | 2/3 | 0/3 | 0/3 |
|  | Road 2 | 3/3 | 0/3 | 0/3 |
|  | Road 3 | 3/3 | 3/3 | 0/3 |
|  | Road 4 | 3/3 | 0/3 | 0/3 |
|  | Cattle | 3/3 | 0/3 | 1/3 |
|  | Farm 2 | 2/3 | 1/3 | 0/3 |
|  | Farm 3 | 2/3 | 0/3 | 0/3 |

**Table S2. Summary of standard curves of three fluorescent**

| First set of multiplex PCR | |  |  |  |
| --- | --- | --- | --- | --- |
| Fluor | Efficiency % | Slope | Y-Intercept | R^2^ |
| FAM | 74.89886 | -4.11884 | 43.50005 | 0.965732 |
| HEX | 96.24378 | -3.41535 | 41.00183 | 0.974332 |
| TAMRA | 87.59443 | -3.66006 | 41.65888 | 0.983774 |
| Second set of multiplex PCR | |  |  |  |
| Fluor | Efficiency % | Slope | Y-Intercept | R^2^ |
| FAM | 78.39781 | -3.97789 | 44.23249 | 0.981567 |
| HEX | 90.13505 | -3.58343 | 42.60211 | 0.994312 |
| TAMRA | 96.64432 | -3.40505 | 41.1009 | 0.987812 |
| Third set of multiplex PCR | |  |  |  |
| Fluor | Efficiency % | Slope | Y-Intercept | R^2^ |
| FAM | 77.92214 | -3.99632 | 44.34668 | 0.989219 |
| HEX | 87.88358 | -3.65112 | 42.94908 | 0.998051 |
| TAMRA | 100.8747 | -3.30115 | 40.99088 | 0.995537 |
